# Supplementary material for: Feasibility, acceptability and equity of a mobile intervention for Upscaling Participatory Action and Videos for Agriculture and Nutrition (m-UPAVAN) in rural Odisha, India
Source: PLOS Glob Public Health. 2024 May 14;4(5):e0003206. doi: 10.1371/journal.pgph.0003206 (PMC11093392; doi:10.1371/journal.pgph.0003206)
Supplement: S1 Checklist — (PDF) [file pgph.0003206.s001.pdf]

Inclusivity in global research

PLOS' policy on inclusivity in global research aims to improve transparency in the reporting of research performed outside of researchers' own country or community and ensures that PLOS publications reporting global research adhere to high standards for research ethics and authorship. Authors of relevant research articles may be asked to complete the questionnaire below, which outlines ethical, cultural, and scientific considerations specific to inclusivity in global research. This questionnaire may be requested when researchers have travelled to a different country to conduct research, if research uses samples collected in another country, research with Indigenous populations or their lands, or if research is on cultural artefacts. Researchers travelling to another country solely to use laboratory equipment will not normally be required to complete the questionnaire. However, the questionnaire can be requested at the journal's discretion for any submission – if you have been requested to complete this questionnaire by the PLOS journal you submitted to, please do so.

Please complete the questionnaire below and include this as a Supporting Information file with your manuscript. Note that if your paper is accepted for publication, this checklist will be published with your article in the supporting information files. Please ensure that you reference the checklist in the main body of your manuscript. We suggest adding a subsection 'Inclusivity in global research' to your Methods section and adding the following sentence: "Additional information regarding the ethical, cultural, and scientific considerations specific to inclusivity in global research is included in the Supporting Information (SX Checklist)"

The questions have been designed to be applicable to a wide range of study types, and there are subsections for both human subjects research and non-human subjects research. If any of the questions are not relevant to your research please mark them as "N/A" as appropriate.

Ethical considerations, permits and authorship

This section is applicable to all research types.

Provide details as to who granted permissions and/or consent for the study to take place in the Methods section of your manuscript. This should include the names of **all** ethics boards, governmental organizations, community leaders or other bodies that provided approval for the study. If individuals provided approval refer to these people by their role or title but do not list their name(s).

Reported on page number: 5

If there were any deviations from the study protocol after approval was obtained please provide details of these changes in the Methods section of your manuscript.

Reported on page number: NA

Did this study involve local collaborators that are residents of the country where the research was conducted or members of the community studied? If you do not have any authors from said communities, please provide an explanation for this below.

Our study included local collaborators who are residents of the country where the research was conducted from the following organisations: Digital Green, Voluntary Association for Rural Reconstruction and Technology (VARRAT), DCOR Consulting Pvt. Ltd, and Ekjut. These collaborators have been included as authors in the manuscript.

Everyone listed as an author should meet PLOS' criteria for authorship and all individuals who meet these criteria should be included in the author byline, rather than the acknowledgements. For further information please see the journal's Authorship Policy.

Human subjects research (e.g. health research, medical research, cross-cultural psychology)

Did you obtain written informed consent from a representative of the local community or region before the research took place? How did you establish who speaks for the community? Details of written informed consent obtained from study participants should be reported separately in the Methods section of your manuscript.

This study, initiated during the COVID-19 lockdown, was built on prior work in the study community as part of the UPAVAN trial, where village leaders provided their informed consent for the participation of villages in the trial. For the present study, which is an extension of the trial, we engaged with target local community groups and explained the purpose and objectives of the research project and their envisaged role and participation whilst following local regulations regarding COVID-19 safety. This engagement was facilitated by a local NGO and project collaborator, VARRAT, who has extensive experience working with the participating communities, a strong presence and rapport among community members, and rich contextual knowledge. For phone surveys, we sought informed verbal consent from participants, which was recorded and documented by DCOR. For qualitative interviews administered in person, we sought written (signature or thumbprint) informed consent from participants. All ethics protocols were reviewed and approved by the local ethics committee.

How did members of the local community provide input on the aims of the research investigation, its methodology, and its anticipated outcome(s)?

Local collaborators of this study, including members from VARRAT, Digital Green, DCOR Consulting Pvt. Ltd, and Ekjut, were involved in all aspects of the research process, including the investigation's aims, methodology, and anticipated outcomes.

The components of the pilot intervention were also driven by community demand. m-UPAVAN was an adaptation of the UPAVAN interventions, which included a participatory video-based approach to agriculture extension. Community feedback was actively elicited through in-built implementation systems, to ensure that the intervention content was driven by community demand and preference. This same content, but adapted for viewing and listening on mobile phones, was used in the m-UPAVAN pilot intervention. Our pilot intervention also incorporated learnings from the UPAVAN interventions mixed-method process evaluation, which sought to understand participants' experiences and perceptions of the UPAVAN interventions through qualitative interviews, group discussions, and case studies among local intervention facilitators and intervention participants.

When engaging with the local community, how did you ensure that the informed consent documents and other materials could be understood by local stakeholders?

Local project partners, DCOR Consulting Pvt. Ltd, co-developed the informed consent documents with the UK-based team, and with input from the local implementing team, VARRAT. DCOR has conducted extensive work, particularly data collection, within our study setting. Further, the UK-based Principal investigator of this study, Suneetha Kadiyala, had over 10 years of experience (at the time of the study) conducting research within the local community and possesses comprehensive contextual understandings to ensure the informed consent documents were understood by local stakeholders. The local ethics committee went through and and approved the informed consent documents.

Members of DCOR Consulting Pvt. Ltd, were responsible for translating the informed consent documents from English Language to Odia Language (the local language). We then back-translated the forms to ensure accuracy, and piloted them before use to ensure they could be easily and well understood by prospective participants. A declaration 'Statement of Accuracy of Translation' was signed and provided to the local ethics committee, Sigma Institutional Review Board.

Will the findings of the research be made available in an understandable format to stakeholders in the community where the study was conducted (e.g. via a presentation, summary report, copies of publications, etc.)? Please provide details of how this will be achieved.

Summary reports and presentations of findings were created and shared through several meetings throughout the duration of the project and since its completion with local collaborators, such as VARRAT and Ekjut. These local collaborators continue to work in the communities or other communities nearby where the study was conducted.

Non-human subjects research using specimens/ animals collected as part of the study, or those housed in archival collections. Examples include archaeology, paleontology, botany and zoology.

Did the permission you obtained from a local authority to perform the study include an agreement on access to outputs and benefit sharing? This may include procedures to enable fair distribution of the benefits and resources arising from the research performed. Please include any details of Prior Informed Consent and Benefit Sharing Agreements obtained. These may be required by field-specific regulations, for example the Convention on Biological Diversity (CBD) and the associated Nagoya Protocol.

N/A

If the material used in your study was imported, please A) provide the year it was imported and B) indicate whether permits were obtained to import/export the materials used, C) provide details of any permits obtained. If this information is not available, please indicate this.

N/A

If you used archival specimens, please state how the material used in your study was acquired by the institute it is held in and provide details of any permits obtained for the original excavations/ sample collection. If this information is not available, please indicate this.

N/A

How was the potential cultural significance of the materials collected in your study to local communities considered in your research design? Were Indigenous peoples and/or local researchers and institutions involved with archaeological excavations / collection of specimens? If so, please provide a description of their involvement.

N/A

If your manuscript includes photographs of human remains please indicate whether authors obtained permission from descendants or affiliated cultural communities to do so.

N/A
