# Supplementary material for: Feasibility, acceptability and equity of a mobile intervention for Upscaling Participatory Action and Videos for Agriculture and Nutrition (m-UPAVAN) in rural Odisha, India
Source: PLOS Glob Public Health. 2024 May 14;4(5):e0003206. doi: 10.1371/journal.pgph.0003206 (PMC11093392; doi:10.1371/journal.pgph.0003206)
Supplement: S1 Table — Participants in UPAVAN study areas who confirmed they participated in the UPAVAN interventions were administered open-ended questions regarding preferences for m-UPAVAN vs UPAVAN. All mothers, besides those belonging to a household without a phone, were recruited from those who participated in monthly monitoring surveys. (DOCX) [file pgph.0003206.s002.docx]

| Characteristics | N (%) |
| --- | --- |
| Social group |  |
| Scheduled Caste | 7 (18.4) |
| Scheduled Tribe | 18 (47.4 |
| Other backward Caste | 13 (34.2) |
| Smartphone in household | 18 (47.2) |
| Mother owns a phone | 24 (63.1) |
| No phone in the household | 5 (13.2) |
| Known participation in the UPAVAN interventions | 7 (18.4) |
| UPAVAN study area |  |
| Intervention areas | 29 (76.3) |
| Control area | 9 (23.7) |
