# Supplementary material for: Feasibility, acceptability and equity of a mobile intervention for Upscaling Participatory Action and Videos for Agriculture and Nutrition (m-UPAVAN) in rural Odisha, India
Source: PLOS Glob Public Health. 2024 May 14;4(5):e0003206. doi: 10.1371/journal.pgph.0003206 (PMC11093392; doi:10.1371/journal.pgph.0003206)
Supplement: S2 Table — (DOCX) [file pgph.0003206.s003.docx]

| **Topic** | **Video shared date** | **YouTube link of video** |
| --- | --- | --- |
| Benefits of hand washing with soap | 17/03/21 | <https://youtu.be/49hfeBFsxQk> |
| Cultivation of different species of Amaranthus for diet diversity year-round | 24/03/21 | <https://youtu.be/gS6O9Hj3SOA> |
| Growing Little gourd for nutrition and income | 31/03/21 | <https://youtu.be/l0VzsdjGJbg> |
| Growing Indian spinach for nutrition | 07/04/21 | <https://youtu.be/IHJiNwd8KrM> |
| Importance of first 1000 days | 14/04/21 | <https://youtu.be/CVhCMI3JM2Q> |
| Prevention and control of diarrhoea | 21/04/21 | <https://youtu.be/FMuTp3lIPvE> |
| Nutrition during pregnancy | 28/04/21 | <https://youtu.be/433G756upLI> |
| Nutrition during lactation | 05/05/21 | <https://youtu.be/uvx8ay_sP2o> |
| Paddy straw mushroom cultivation for income | 12/05/21 | <https://youtu.be/Tn-v7WGhux4> |
| Maternal workload – Impact of and strategies to reduce workload | 19/05/2021 | <https://youtu.be/rxsOW78UlVw> |
| How to practice chicken farming. | 26/05/2021 | <https://youtu.be/DmAwG29eddc> |
| Exclusive breastfeeding | 02/06/2021 | <https://youtu.be/ocg5pNKJVlY> |
| Introducing complementary feeding | 09/06/2021 | <https://youtu.be/gbGgpgdqke4> |
| Age-appropriate complementary feeding (6 - 9 months) | 16/06/2021 | <https://youtu.be/uYT_wVLVBU4> |
| Age-appropriate complementary feeding (9 - 12 months) | 23/06/2021 | <https://youtu.be/XSppyzJsSRc> |
| Age-appropriate feeding for children (12 - 24 months) | 30/06/2021 | <https://youtu.be/lEK6PkR05do> |
| Benefits of line transplanting and seed treatment | 07/07/2021 | <https://youtu.be/xmu63QZ3W1s> |
| Nursery management and line transplanting | 14/07/2021 | <https://youtu.be/2L89QFZIEmw> |
| Feeding of a sick child during illness | 21/07/2021 | <https://youtu.be/gIJmx1OGaXY> |
| Goat farming - vaccination and shed management | 28/07/2021 | <https://youtu.be/JtutQy5JtmU> |
| Recipes for dietary diversity for complementary feeding | 04/08/2021 | <https://youtu.be/sPjx87XW2zU> |
| Preparation and application of pot manure | 11/08/2021 | <https://youtu.be/41cY8JgiBCU> |
| Building a kitchen garden for nutrition | 18/08/2021 | <https://youtu.be/8IGr9cqyVKM> |
| Papaya + banana + drumstick cultivation for consumption | 25/08/2021 | <https://youtu.be/Z6my5qB0zMw> |
| Reinforcing dietary diversity | 01/09/2021 | <https://youtu.be/PCMZHemoFs0> |
| Growing carrots for nutrition | 08/09/2021 | <https://youtu.be/wH07--HEcYI> |
| Nutrition and care of a new born and an underweight child | 16/09/2021 | <https://youtu.be/i5DQFVbDxpE> |
| Jaupoka control in cowpea | 22/09/2021 | <https://youtu.be/vCNcp7RNDjM> |
| Nutrient management for the milking stage of Paddy | 29/09/2021 | <https://youtu.be/vsJFId3Z21Q> |
