# Supplementary material for: Feasibility, acceptability and equity of a mobile intervention for Upscaling Participatory Action and Videos for Agriculture and Nutrition (m-UPAVAN) in rural Odisha, India
Source: PLOS Glob Public Health. 2024 May 14;4(5):e0003206. doi: 10.1371/journal.pgph.0003206 (PMC11093392; doi:10.1371/journal.pgph.0003206)
Supplement: S1 Appendix — (DOCX) [file pgph.0003206.s004.docx]

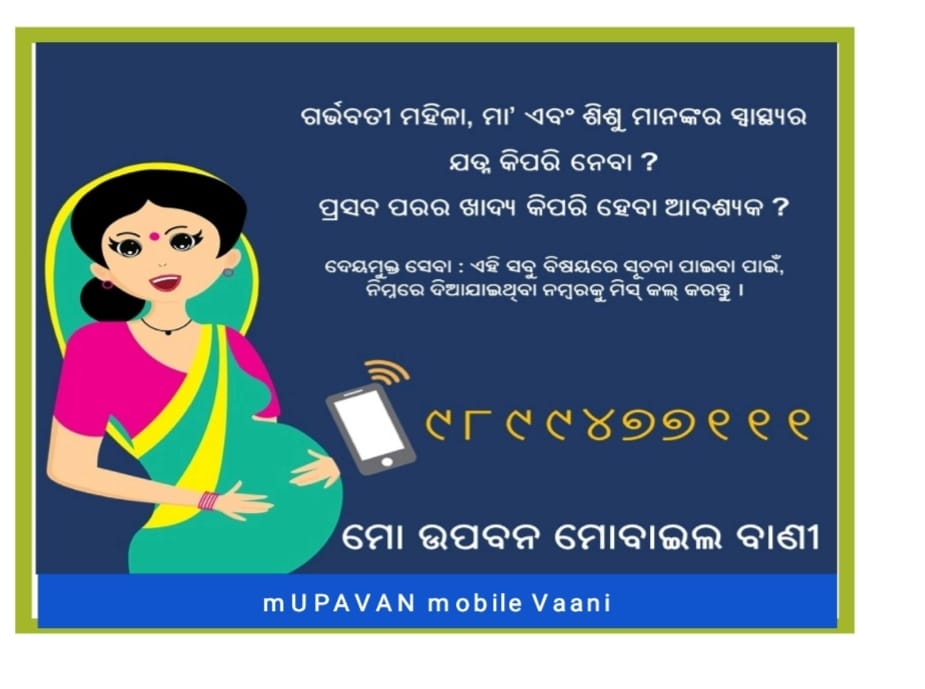


In the English language, the Odiya text on the poster translates to: *“How can we ensure the health and well-being of pregnant women, mothers, and children? What are the suggested dietary choices for women during the postpartum period? Free Service: To obtain complete information on the subjects mentioned above, simply give a missed call to the number provided below”*.
