# Supplementary material for: Feasibility, acceptability and equity of a mobile intervention for Upscaling Participatory Action and Videos for Agriculture and Nutrition (m-UPAVAN) in rural Odisha, India
Source: PLOS Glob Public Health. 2024 May 14;4(5):e0003206. doi: 10.1371/journal.pgph.0003206 (PMC11093392; doi:10.1371/journal.pgph.0003206)
Supplement: S2 Appendix — (DOCX) [file pgph.0003206.s005.docx]

***NOTE TO THE INTERVIEWERS BEFORE STARTING THE INTERVIEW:***

- This is a guide and NOT A questionnaire.
- Treat the interview as a discussion and not a back-and-forth with questions and answers
- Each question is a topic (from now on, ‘topic’), and the points below are only to ensure that these topics are covered in the conversation.
- When you start each topic, use terms like “*please tell us”,* “*could you explain”*, “*how”, and “why”*. Avoid phrasings that give you just yes or no answers (for example, did you, are you, were you, etc?)
- Once you start the topics, let the mother answer fully. Use the probes if she did not get to the topics we needed to focus on.
- Please do not read out the topic first and then right away launch into probes; do not treat probes as sub-questions. Probes are guides or key points for discussion.

***Module 1****: Explore how the informant engages with the mobile phone in the household and how the intervention fits in with their usual usage, social and cultural norms, and the overarching acceptability of digital health interventions*

1. Ask about the mother’s household structure and composition. Use the following probes to guide your conversation:
   1. Tell us who is in your family.
      - How many children and how old are they?
      - How many adults?
      - How are you related to them (adults and children)?
2. Understand phone ownership and access. Use the following probes to guide your conversation:
   1. Tell us how many phones are in your household.

Check which phones are smart and which ones are not smartphones (touch screen vs. small /button mobile phone)

- 1. Who owns the phones(s)?
     - Probe who in the household uses smartphones vs. non-smartphone (small /button mobile phones)
     - Confirm whether the mother owns a phone or not and find out what kind of phone it is
  2. Who usually uses the phone(s)?
     - Ask about things in and outside the household, e.g., do people in this household lend their phones to others? If yes, to whom and what?

*If the mother does not personally own a phone: (note: Here we are trying to understand if she has autonomy in using someone else’s phone)*

- 1. Please explain how you access a phone, if at all.
     - Whose phone(s) do you access? Probe about household members’ phones, neighbours, relatives, friends or other’s phones?
     - How easy or difficult is borrowing someone else’s phone?
     - Tell us the process of accessing the phone. (for example, does she need permission, can she do this only at certain times of the day, why?)
     - Does the phone owner have to approve what she uses it for?
     - Can she use it in private?
     - How often do you access someone else’s phone?
  2. What kind of phone are you able to access, a smartphone or a regular phone?

1. Understand the informant’s barriers to phone usage. Use the following probes to guide your conversation:
   1. Tell us how easy or difficult it is to use the phone and access data
      - What about data costs?
      - What about data coverage/network?
      - How often do you/your HH members change plans and numbers?

*We do ask some of the topics below in topic 2 above; this is another chance to get any more information.*

- - - Tell us about accessing the phone only during a specific time of the day.
    - What about any permissions needed to use the phone?
    - What about permissions for what you can or cannot use the phone for?
  1. What other factors affect how and what you use the phone for?
     - Explore if there are any gender, cultural or any other barriers or social norms associated with what she could use the phone for or in accessing the phone itself

1. Understand the mother’s use of the phone in the last 6 months. Use the following probes to guide your conversation and get information about how the mother is situated within the household and the community:
   1. Could you tell us what activities you usually use the phone for and how long you spend on it for each activity?

- If she accesses a smartphone, tell us what apps or sites (social media) you browse and for what purpose. Can you give some examples?
- Tell us about the WhatsApp account you use.
  - For example, did you open or use another’s WhatsApp account?
  - Tell us if you ever Chat with anyone using WhatsApp.
    - What about health interventions?
      - From whom, how often, and how long?
      - What about contacting the community health workers? For example, AWW, ASHA and other NGO workers
        - From whom, how often, and how long?
    - What about food and nutrition information (such as your own and your children’s food and nutrition)
      - From whom, how often, and how long?
      - What about contacting the community health workers? For example, AWW, ASHA and other NGO workers
        - From whom, how often, and how long?
    - What about agriculture-related activities?
      - From whom, how often, and how long?
      - What about contacting community extensions workers or from NGOs?
        - From whom, how often, and how long?
    - What about selling / marketing of agriculture produce?
      - From whom, how often, and how long? Probe about FPOs and contact with companies
    - Any SHG-related activities?
      - From whom, how often?
    - What about education for you or your family members?
      - From who, how often, and how long?
    - Any other activities?
  1. Are there any other activities or ways you wish you could use the phone (but are unable to?)
     - Please could you tell us more about this?

E.g., why is this a want/need/priority you,

- - - Tell us why you are unable to get this information via your mobile?
      - Probe about is it because there are no programmes for thus or because she has limited access to phones, or if she needs permission to get this information via mobile
  1. Please could you tell us if and how your phone usage has changed since COVID-19?
     - Why do you think it has changed?

***Module 2:*** *Assess the acceptance of the underlying principles of the intervention, receiving health and agriculture messages via WhatsApp and IVR, and how this influences participation*

1. Understand whether the informant is familiar with m-UPAVAN. Use the following probes to guide your conversation:
   1. Have you heard about m-UPAVAN?

Please describe the m-UPAVAN intervention.

*NOTE TO INTERVIEWER: Here we are trying to understand whether the mother knows it’s the WhatsApp messages and IVR we send and the two major topics we cover (agriculture and nutrition)*

- 1. How did you hear about the intervention, and from whom?
  2. What were your thoughts when you first heard about the intervention?
     - How and why were you interested or not interested?
  3. Did you share about the intervention with your household members? What did your household members think?
     - What was their reaction?
     - Did they think it would be useful or not, and why?
  4. Tell us about conversations about the intervention when you first heard about it with:
     - Family members
     - With friends/community members
  5. In the case you were using another’s phone, tell us about his/her reaction when you shared with them that the m-UPAVAN videos or IVRS messages will be sent to their phones. Did they show interest?
  6. Tell us about your reaction when you knew that you had to use others' phones for IVR or their WhatsApp account for the m-UAPAVAN intervention.

*NOTE TO INTERVIEWER: d-f: one of the objectives of d-f is that we are trying to get to the priorities, values, and belief systems of the household*

***Module 3:*** *Assess the acceptance of the delivery platform for the intervention, Whatsapp and IVR, as well as perceptions of the strengths and weaknesses of using that approach and areas it could be improved*

1. Explore the informant’s interaction with the intervention (Whatsapp, IVR messages on nutrition and agriculture). Use the following probes to guide your conversation:

*NOTE TO INTERVIEWER: Please ask the informant to be specific as to whether they are referring to videos or IVR messages and also whether to agriculture or health messages.*

- 1. Please describe m-UPAVAN activities…what aspects of the intervention did you participate in? Probe for the below and about agriculture and nutrition
     - Watching videos on WhatsApp (and in relation to agriculture, nutrition)
     - WhatsApp groups chats and other activities like sharing photos (and in relation to agriculture, nutrition)
     - IVR line (and in relation to agriculture, nutrition)
  2. Tell us how easy or difficult it is to access each m-UPAVAN activity, and why?
  3. Can you please tell us how you usually access these activities? Probe the below.

Was it via your own phone, another household member’s phone, a neighbour’s, CSPs phone?

- - - Did you require permission before engaging in these activities, or could you do so when you wanted?

Overall, in the last six months, how many phones or WhatsApp accounts did you use to access the videos or IVR messages?

- 1. Could you please describe experiences with watching videos/listening to IVR lines, and contributing to WhatsApp chat? [ask separately for each that she mentioned in 6a and in relation to both agriculture and nutrition messages)]
     - What did you like or not like, and why?
     - Please describe how other household members participate in these activities.
       - , e.g., did they watch the videos or listen to the IVR messages with you or separately?
         - how often and with whom?
         - *Did your household members watch both* agriculture and nutrition videos?
       - What did your household members think of these messages?
         - Tell us the topics they liked to watch videos about and what they did not prefer to watch

- - - Do you think it’s important for other household members to engage with the activities, and why/why not?
    - What other factors affected (positively/negatively) you and your household members' participation in the activities? Why/why not?
      - Probe for times of year or stages of life when they might start engaging more, e.g., husband migrating, pregnancy, evenings when the family is together, etc
    - How did you find watching the m-UPAVAN videos compared to other agriculture and nutrition videos you received and watched? Please provide some examples of whether you find them interesting or not.

*If the informant mentioned participating in m-UPAVAN WhatsApp chats…*

- 1. Can you please describe your participation in these chats?
     - What was the conversation about?
     - What motivated you to chat on WhatsApp about the intervention?
     - Would you have liked to have chatted more on WhatsApp about the intervention? What would have helped you do so?
  2. How does your access to a phone affect your participation in the intervention?
     - Do you feel you can access a phone enough to participate fully/as much as you’d like?
     - Can you describe a time when your phone access prevented you from participating when you wanted to?
       - What happened and has it been resolved?
  3. What could m-UPAVAN do to improve your participation in the intervention?

1. Understand how the CSP's role and how the mother felt the CSP helped the informant engage with the intervention.
2. Have you heard of CSPs? Please describe what you understand about CSP’s role.

1. Tell us about your contact with the CSP? Also, *probe* for:
   - Please tell us how many times the CSP contacted you in person or over the phone in the last six months?
   - Please describe the discussion between you and the CSP when the CSP contacted you. Please give us as many examples as you can think of.

*Probe further, if needed, on the following to understand the interaction with CSPs:*

- - How did and how often the CSP encourage you to watch the videos or listen to the IVR message?
    - Please give examples and recall your conversation with the CSP in detail.
  - Tell us your interactions, if any, with the CSP regarding WhatsApp group chat?
  - Did the CSP help reinforce/remember what was discussed in the video or IVR message after you had watched or listened?
    - If yes, How? Please describe with examples

1. Do you have any ideas on how the role of the CSP could be improved to increase your engagement with m-UPAVAN?

***Module 4***: *Assess the acceptability of the content of the intervention*

1. Understand what the informant thinks about the content of the intervention. Use the following probes to guide your conversation:
   1. What aspects of agriculture, nutrition, and health were the most and least important to you that the intervention addressed in m-UPAVAN?
      - Why is this aspect important to you?
        - Repeat for all aspect mentioned and probe if she has not mentioned nutrition or agriculture
      - How about your other household members?
   2. Which topics of the intervention were new and interesting for you, and why?
   3. Which topics did you think were not relevant or interesting for you, and why?
   4. What would have been more useful to learn about for the mother and her family?
      - How does she think this would benefit her health/her child’s health, or her family?
   5. Which m-UPAVAN promoted practices could you put into practice? And why? Probe for
      - Agriculture (note: the m-UPAVAN messages took into account seasonality, land and water. If the respondent says these are the barriers, ask probe about the specific issues and what could have helped?)
      - Nutrition
      - Probe for intra-household dynamics (for example, with spouse, in-laws, etc and try to ask if these people saw the videos, IVR, what they thought, etc).
   6. Beyond nutrition and agriculture, could you think of any other ways in which m-UPAVAN affected you or your family members? probe for:
      - Process of using the phone, apps, accessing the internet etc
      - Communication, in general, or specific to agriculture or nutrition, with other household members

***Module 5:*** *Explore the informant’s experiences watching the videos or listening to the IVR messages and the discussions and activities in the household that followed this to understand the potential the intervention has to be effective.*

1. Explore the processes that occur after the informant watches a video or listens to an IVR message. Use the following probes to guide your conversation:
   1. Can you explain what you usually do after a new video or IVR message is released each week?
      - Did you have to organise watching on the phone, get any permissions, or discuss timings
      - Watch alone, wait for other family members/friends, or watch first alone and then share with others if you think it is interesting/relevant.
   2. What happened after you watched the video or listened to the IVR message?

*If she watched or listened with others:*

- - - Were there any further discussions with other household members about these videos?
      - Who did you usually have these discussions with?
    - What kind of discussion do you have, and with whom?
    - Can you please, with examples, describe the discussions that usually took place after you watched a video or listened to an IVR message? *Probe*:
      - Discussions about what you liked/did not like about the messages
      - Discussions on which messages were relevant for your household situation?
      - Whether you discussed adopting the practices? Give examples.
      - Were there more /less discussions after agriculture of nutrition videos? How? And why?
    - What did you think about the skills/behaviours promoted?
    - What did others watching with you think about the skills/behaviours promoted? Did you agree or disagree about whether to try out the practice?
      - Give a few examples of what you agreed about and what you disagreed about and why.
      - When you disagreed with other household members, why did you have differences of opinion and how was this resolved?

*If she watched alone…*

- - - Did you share any of the information, knowledge or experience learned from the video or IVR message with anyone, either within or outside her household, and why/why not?

*If yes…*

- - - Who did she share the information with, and why?
    - Whether it was easy or hard to share, and why;
    - What did others you shared information with think about the skills/behaviours promoted? Did you agree or disagree about whether to try out the practice?
      - Give a few examples of what you agreed about and what you disagreed about and why.
      - When you disagreed with other household members, why did you have differences of opinion and how was this resolved?
        1. Were there instances where you thought it was not possible to do a m-UPAVAN promoted practice and others persuaded you? How?
        2. Were there instances where you thought you could adopt the practices, but others dissuaded you? How and why?

1. Understand whether the mother and/or her household members have considered adopting new agriculture or nutrition practices promoted in m-UPAVAN or whether they have adopted anything new.
   1. Have you thought about adopting any of the nutrition of agriculture practices m-UPAVAN prompted?

NOTE FOR INTERVIEW: Here we are trying to get to if the mother and /or household members at least *thought* of these adopting the m-UPAVAN promoted practices. It is not about doing them (this is in the next question).

- - - Which practices did you consider/think of and why?
    - What do other family members think about this?
    - What practices were easy for you or your household to consider?
    - What nutrition and agriculture practices do you think would be challenging for you and your household to adopt, and why?
  1. Have you adopted nutrition behaviours or agriculture practices since learning about them in the m-UPAVAN?
     - If yes, which?
     - Please can you talk us through how you went about doing this?
     - What made putting adopting this practice easy or challenging?
     - How has this practice positively or negatively affected you/your child/your family?

M***odule 6:*** *NOTE TO INTERVIEW: THIS MODULE IS ONLY FOR INFORMANTS WHO PARTICIPATED IN BOTH INTERVENTIONS (m-UPAVAN and UPAVAN).*

*Access the relative advantages/disadvantages of videos or IVR messages being delivered to mobile phones or watching in women’s self-help groups. First, familiarise the informant with the UPAVAN trial and distinguish the key differences between the UPAVAN (watching video dissemination on nutrition and agriculture practice in women’s SHGs and m-UPAVAN (receiving the video through WhatsApp groups on mobile phones or dialling the IVR line from a mobile phone)*

1. Please tell us how UPAVAN is currently influencing your agriculture and nutrition practices?
   1. Have you adopted any agriculture and nutrition practices during UPAVAN?
      - Please describe which ones for agriculture and which ones for nutrition
   2. Which of these practices have you continued with and for how long after UPAVAN?
      - How long did you adopt agriculture and nutrition practices promoted by UPAVAN? Which ones? Probe for agriculture and nutrition
      - Are you *currently* practicing them? Describe which ones? Probe for agriculture and nutrition
      - If no, why did you stop adopting these practices? Please describe?
        - What would have helped you continue with these practices
   3. Did you take up any new practices that UPAVAN promoted, since March 2020 (since COVID started?)
   4. Tell us if your participation in UPAVAN helped with coping during COVID. How and why?
2. Understand which intervention, UPAVAN or m-UPAVAN, the informant preferred. Use the following probes to guide your conversation:
3. Having participated in both UPAVAN and m-UPAVAN, what aspects of watching videos in SHGs do you prefer compared to having the videos delivered to mobile phones or listening to the IVR line and vice versa? Probe for:
   - - What made it more engaging, and why?
     - What was more enjoyable, and why? *Probe to understand which aspects of UPAVAN-m-UPAVAN they prefer and why*
       - Video long story format vs. short videos or IVR voice message only
       - Meeting other women and social engagement vs. watching alone or with family members
         - For PLA women, PLA activities, e.g., prioritising problems, understanding the cause and effects of problems, identifying feasible strategies, purely discussion-based meetings
       - Collective vs. household level (for example, feeling like the community is there to support you in UPAVAN vs. family members are more likely to engage in m-UPAVAN) *DO NOT LEAD*
       - Nature of the discussion and dialogue in SHG vs. with other household members
       - Phone dependency and access issues vs. not being responsible with phones/data/internet for UPAVAN
     - Which was easier/harder for her to participate in, and why?
       - Permission to attend meetings (UPAVAN) or use a phone (m-UPAVAN)
       - Her time
       - Pregnancy and other childcare
       - Other
     - What was most interesting about either intervention?
4. Did you find the skills and practices discussed in the video disseminated in SHGs (and PLA if the participant is for the UPAVAN PLA arm) easier or harder to understand compared to videos delivered to mobile phones or listening to IVR messages, and why?

After the informant has responded, further probe for:

- - - (UPAVAN arm 3 participants) Participatory nature of UPAVAN intervention, e.g., PLA cycle, games, understanding cause and effects of problems, purely discussion-based meetings
    - Pausing of videos for discussions and questions, home visits
    - m-UPAVAN WhatsApp group chats, re-watching videos, MCQs in group chat,
    - Discussions with other SHG members
    - Discussions with family

1. What is more important to you, watching agriculture and nutrition videos with family members or SHGs at home, and why?
   - - Which family members does she think it most important to watch with, and why?
       - When participating in UPAVAN, who were you mostly likely to share and discuss information
       - In m-UPAVAN, who were you mostly likely to share and discuss information
     - Which intervention facilitated more discussion on the practices and behaviour learned in either intervention and why was this? And with whom>
     - Which intervention facilitated the adoption of practices and behaviour learned in either intervention, and why was this?
       - Also probe about the support they got in adopting practices in UPAVAN vs m-UPAN? (from friends/ other SHG members, household members)

*For women from UPAVAN arm 3 (PLA arms), further probe for:*

- - - Importance of PLA activities in women’s groups, e.g., prioritising problems together, understanding the cause and effects of problems, identifying feasible strategies together, purely discussion-based meetings, learning from others

1. Do you think more other household members are more supportive of adopting new skills and practices in the current intervention (m-UPAVAN) or those learned from UPAVAN, and why;
2. What do you think about information received on mobiles about agriculture and nutrition compared to face-to-face intervention?
   - - Do you trust it more/less, and why?
     - Which motivated you more to try new skills, and why?

*For women from UPAVAN arm 3 (PLA arms), further probe for:*

- - - - PLA activities, e.g., prioritising problems, understanding the cause and effects of problems, identifying feasible strategies, purely discussion-based meetings, shared learning/learning from others' experiences

1. Having participated in both of these interventions…

- Which one would you choose and why?
- Which would you recommend to others, and why?
- How could your preferred intervention be improved?
