# Supplementary material for: Feasibility, acceptability and equity of a mobile intervention for Upscaling Participatory Action and Videos for Agriculture and Nutrition (m-UPAVAN) in rural Odisha, India
Source: PLOS Glob Public Health. 2024 May 14;4(5):e0003206. doi: 10.1371/journal.pgph.0003206 (PMC11093392; doi:10.1371/journal.pgph.0003206)
Supplement: Attachment — Submitted filename: Response to Reviewers.docx [file pgph.0003206.s006.docx]

Feasibility, acceptability and equity of a mobile intervention for Upscaling Participatory Action and Videos for Agriculture and Nutrition (m-UPAVAN) in rural Odisha, India.

*Response to Reviewers*

We want to thank the editor and the reviewer for their time in reviewing and commenting on our manuscript. Based on your feedback, we feel the revision has been enhanced, and we have provided a point-by-point response below.

*Reviewer 1*

1. *Abstract: The authors do not provide the results in a readable and logical way. As a reader it will be great to see the abstract (especially results) written in a phased manner.*

Thank you for this comment. We have revised the abstract to be written in a phased manner and switched to the active voice to improve readability.

2. *Line 163: the role and contribution of Digital Green is somewhat unclear. Did they participate in developing the model for initial UPAVAN. If no, then what qualifies them to be a creator for m-UPAVAN in this case.*
   *The authors previously asserted the composition of UPAVAN's content (line 75-83) with its goals. But this not clear with m-UPAVAN. It seems that m-UPAVAN applies the same principles like UPAVAN but not same content. This distinction has to be clearly stated. The decision to also use the word m-UPAVAN thus becomes questionable.*

Thank you for requesting this clarification, and we apologise that this was not clear in the submitted manuscript. Digital Green was involved in the original UPAVAN interventions in the same capacity as in the m-UPAVAN intervention. Digital Green designed the participatory video-based approach to agriculture extension used in the UPAVAN interventions, and the nutrition-sensitive and nutrition-specific agriculture video content. This same content was used in m-UPAVAN, but shorted to enable sharing and viewing via WhatsApp, and repurposed as audio messages shared via an interactive voice response system. We have added the following text to clarify this:

*Lines 167-172: Digital Green, an international NGO produced the intervention content. Voluntary Association for Rural Reconstruction and Appropriate Technology (VARRAT), an Odisha-based NGO, was responsible for implementing the intervention in m-UPAVAN villages. Ekjut and JSI provided technical assistance. UCL advised on the intervention content and study design. DCOR led the data collection and data coding. LSHTM led the entire study.* These same set of organisations were partners in the UPAVAN trial.

*Lines 188-194: The UPAVAN interventions built upon a participatory video-based approach to agriculture extension designed by Digital Green but made ‘nutrition-sensitive’ (i.e., promoting practices that address the underlying causes of undernutrition and incorporating specific nutrition goals) and integrating nutrition-specific video topics [16]. In m-UPAVAN, Digital Green reproduced videos covering nutrition-sensitive agriculture and nutrition-specific topics based on those used in the original UPAVAN trial but shortened for sharing and viewing via WhatsApp groups.*

3. *The selected rates decided by the study (line 221-240) seem ambiguous. It is expected that such cut-offs will be selected based on literature or a more scientific approach will be used to ascertain the cut-offs.*

Thank you for this important comment. At the time of our study, there was minimal research on adapting nutrition interventions to mobile interventions during the COVID-19 pandemic. Further, there is a lack of scientific consensus and standardised targets that determine interventions feasible and acceptable from the perspective of the community, which is likely attributable to the highly context-dependent nature of these targets.

Therefore, our research and implementation team arrived at a consensus for targets by considering: 1) achievements of the original UPAVAN interventions (exposure to intervention rates ranged from 50-58%), 2) potential logistical constraints (e.g., phone numbers changing frequently, poor network connectivity, damaged or lost phone), and 3) what the implementation team considered realistic, achievable and would warrant further investment, based on their >30 years of experience in implementing development interventions in our study district. Early studies have adopted a similar approach (1,2). Further, our mixed-method approach prevented over-reliance on our target quantitative indicators and ensured a holistic interpretation of the feasibility and acceptability of the m-UPAVAN intervention.

We have added the following text to our manuscript:

*Line 240-250: Our research and implementation team arrived at a consensus for targets by considering: 1) exposure to the original UPAVAN interventions, which ranged from 50-58% [12] and reflect our m-UPAVAN intervention uptake (proportion of women watching or listening to at least one video or audio in the last month) target of >50%; 2) potential logistical constraints, and 3) what the implementation team considered realistic, achievable and would warrant further investment, based on their >30 years of experience in implementing development interventions in our study district. As suggested by Avery and colleagues [17], indicators falling below our prespecified targets would be reviewed by the implementation team to assess whether further investment in the intervention was still warranted but with caution and or/modifications or whether the indicators suggest intractable issues with the intervention that are irremediable.*

References

1. Meiksin R, Crichton J, Dodd M, Morgan GS, Williams P, Willmott M, et al. A school intervention for 13- to 15-year-olds to prevent dating and relationship violence: the Project Respect pilot cluster RCT. Public Health Research. 2020 Apr 1;8(5):1–338.
2. Ponsford R, Bragg S, Meiksin R, Tilouche N, Van Dyck L, Sturgess J, et al. Feasibility and acceptability of a whole-school social-marketing intervention to prevent unintended teenage pregnancies and promote sexual health: evidence for progression from a pilot to a phase III randomised trial in English secondary schools. Pilot Feasibility Stud. 2022. 2022 Mar 4;8(1):52.doi: 10.1186/s40814-022-00971-y.

Journal Requirements

1. We have uploaded a complete version of our questionnaire on inclusivity in global research as *Supporting Information* (S1 Checklist) with our resubmission and added a section on ‘Inclusivity in Global Research’ to our methods (Line 311-313).
2. We have made the following changes/updates to our reference list:

Reference 2: The webpage for this reference is no longer accessible. This reference has been replaced with a relevant current journal article.

Reference 4: The initials of the surname and forename were the wrong way around, and this error has now been rectified.

Reference 6: The abbreviated journal name was incomplete and has now been corrected.

Reference 13: The webpage for this reference is no longer accessible. This reference has been updated, and an active webpage address provided.

Reference 17: A new reference has been added in response to a reviewer's comment.

Reference 31: The first author’s name for this reference was missing and has now been added.
