# Supplementary material for: Feasibility, acceptability and equity of a mobile intervention for Upscaling Participatory Action and Videos for Agriculture and Nutrition (m-UPAVAN) in rural Odisha, India
Source: PLOS Glob Public Health. 2024 May 14;4(5):e0003206. doi: 10.1371/journal.pgph.0003206 (PMC11093392; doi:10.1371/journal.pgph.0003206)
Supplement: Attachment — Submitted filename: Reviwers comments_UG.doc [file pgph.0003206.s007.doc]

Accept with minor revision

Feasibility, acceptability and equity of a mobile intervention for Upscaling Participatory Action and Videos for Agriculture and Nutrition (m-UPAVAN) in rural Odisha, India

Suggestions:

1. Thank you for coming up with an excellent intervention. I would like to see a bit more analysis, especially the authors’ perspectives on how to overcome digital poverty among rural women of LMICs. Any future direction or research ideas other than mixed kind intervention (face to face and digital) will be a great addition in the manuscript.
2. You mentioned about bias of socio-economic hierarchy in selecting participants. Can you provide suggestions regarding overcoming this biases in future digital interventions?
3. Methodology section is a great read. Please add your reflexivity statements as researchers working on rural context and during telephonic interactions with the participants.
