# Supplementary material for: Feasibility, acceptability and equity of a mobile intervention for Upscaling Participatory Action and Videos for Agriculture and Nutrition (m-UPAVAN) in rural Odisha, India
Source: PLOS Glob Public Health. 2024 May 14;4(5):e0003206. doi: 10.1371/journal.pgph.0003206 (PMC11093392; doi:10.1371/journal.pgph.0003206)
Supplement: Attachment — Submitted filename: Response to Reviewers.docx [file pgph.0003206.s008.docx]

**Feasibility, acceptability and equity of a mobile intervention for Upscaling Participatory Action and Videos for Agriculture and Nutrition (m-UPAVAN) in rural Odisha, India.**

*Response to Reviewers*

We thank the editor and the second reviewer for reviewing and commenting on our revised manuscript. We have addressed the points raised and provided a point-by-point response below. We feel these additional suggestions have considerably strengthened our manuscript.

**Reviewer 2 comments:**

1. Thank you for coming up with an excellent intervention. I would like to see a bit more analysis, especially the authors’ perspectives on how to overcome digital poverty among rural women of LMICs. Any future direction or research ideas other than mixed kind intervention (face to face and digital) will be a great addition in the manuscript.

Thank you for this important suggestion. We have expanded on future interventions and research recommendations, especially related to digital inequality and m-Health interventions, by adding the following paragraph to our discussion:

*Line 710-*721: *Consequently, our study's findings underpin the necessity of addressing inequities in phone access and use to optimise mHealth interventions' potential in low- and middle-income settings. Training and capacity building on phone and mobile app usage in rural communities could help increase the uptake of mHealth interventions and alleviate gender-based social norms in phone usage [42]. Many policy initiatives are being implemented across low and middle-income settings [43,44], including in India [45,46], to improve access to digital technologies in rural and disadvantaged communities. Future mHealth interventions and policy approaches should work closely with relevant initiatives to increase their uptake and effectiveness. mHealth interventions that incorporate a microcredit component to help certain individuals purchase phones may also be a feasible approach to reducing inequities in mHealth [42]. Future research must explicitly evaluate the effects of digital inequality on the efficacy and equity of future mHealth interventions.*

2. You mentioned about bias of socio-economic hierarchy in selecting participants. Can you provide suggestions regarding overcoming this biases in future digital interventions?

Thank you for this suggestion. We have added the following text to our discussion:

Line 728-732*: Whilst unfeasible at the time of our study due to COVID-19 restrictions, evaluations of future digital interventions should aim to use the most inclusive methods available for recruiting and interviewing participants to overcome these biases, such as in-person interviews or using on-the-ground resource persons to help facilitate the participation of harder-to-reach groups in mobile surveys.*

3. Methodology section is a great read. Please add your reflexivity statements as researchers working on rural context and during telephonic interactions with the participants.

We agree that including a reflexivity statement in our manuscript is important. We have added the following text to our methods section. Please note that telephonic interactions were only used to collect quantitative data, which was carried out by a local team of trained data collectors, whilst qualitative data were collected using in-person interviews.

Line 313-320: *This research was primarily undertaken by academic researchers based in the UK and researchers based in and from India. These researchers all have first-hand-contextual experience of the study setting and academic knowledge that likely preconditioned beliefs related to participant realities, which may have influenced interactions with participants during qualitative interviews and interpretations of the data. By acknowledging the diverse positionalities and perspectives within our research team and in relation to our study participants throughout the research processes, we strived to ensure a respectful representation of the lived experiences of the study participants.*
